# Supplementary figures and images for: ENQUIRE automatically reconstructs, expands, and drives enrichment analysis of gene and Mesh co-occurrence networks from context-specific biomedical literature
Source: PLoS Comput Biol. 2025 Feb 11;21(2):e1012745. doi: 10.1371/journal.pcbi.1012745 (PMC11844901; doi:10.1371/journal.pcbi.1012745)

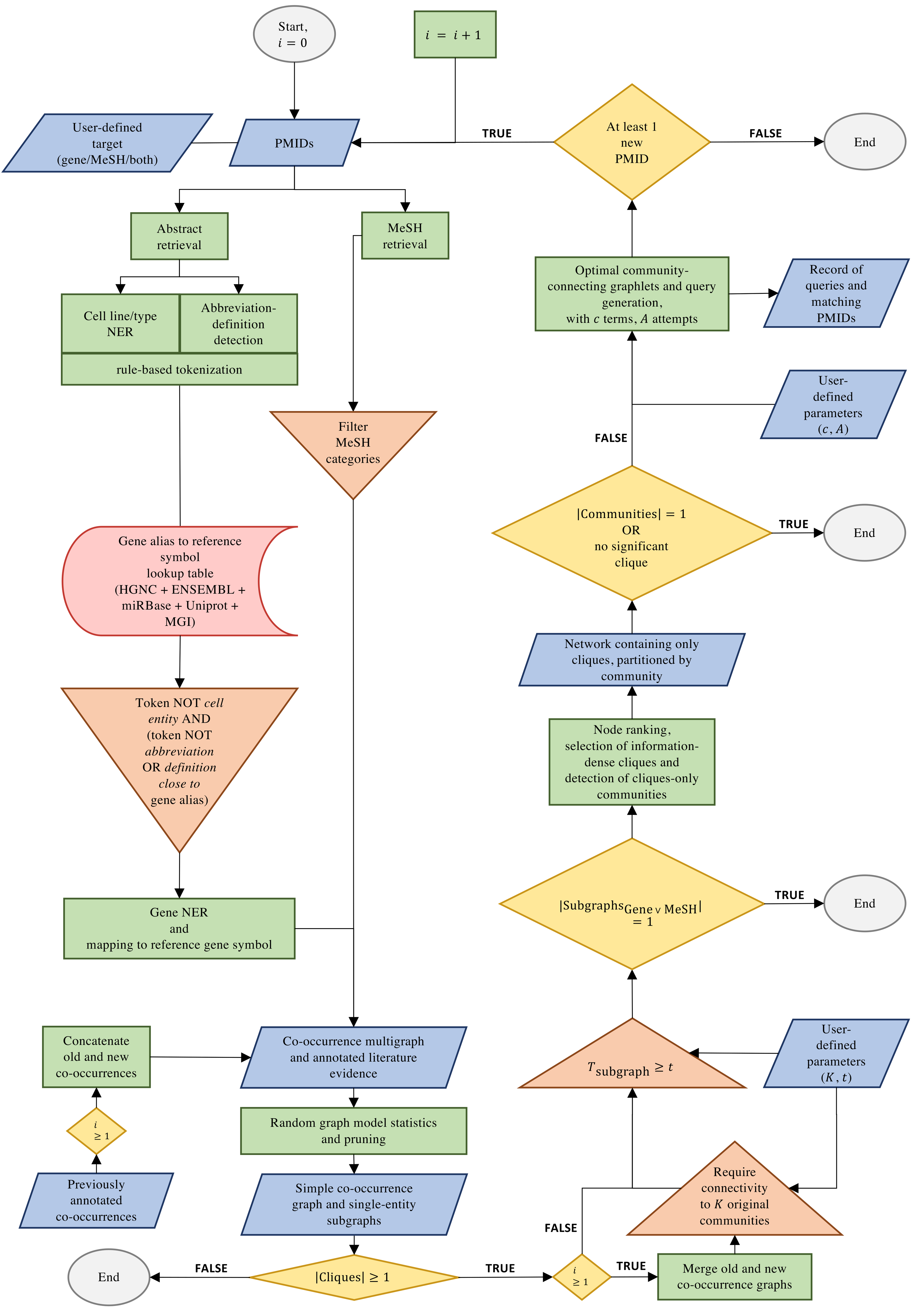

Supplement: S1 Fig — The pipeline’s schematics is described with respect to start and end points (grey ellipses), input, parameters, and generated data (blue parallelograms), algorithms (green rectangles), filtering (red triangles), pre-computed data (pink halfpipes), and branching points (yellow diamonds). NER: named-entity recognition. PMID: PubMed identifier. MeSH: Medical Subject Heading. Detailed explanation of the parameters and algorithms is provided in the main text. (TIF) [file pcbi.1012745.s002.tif]

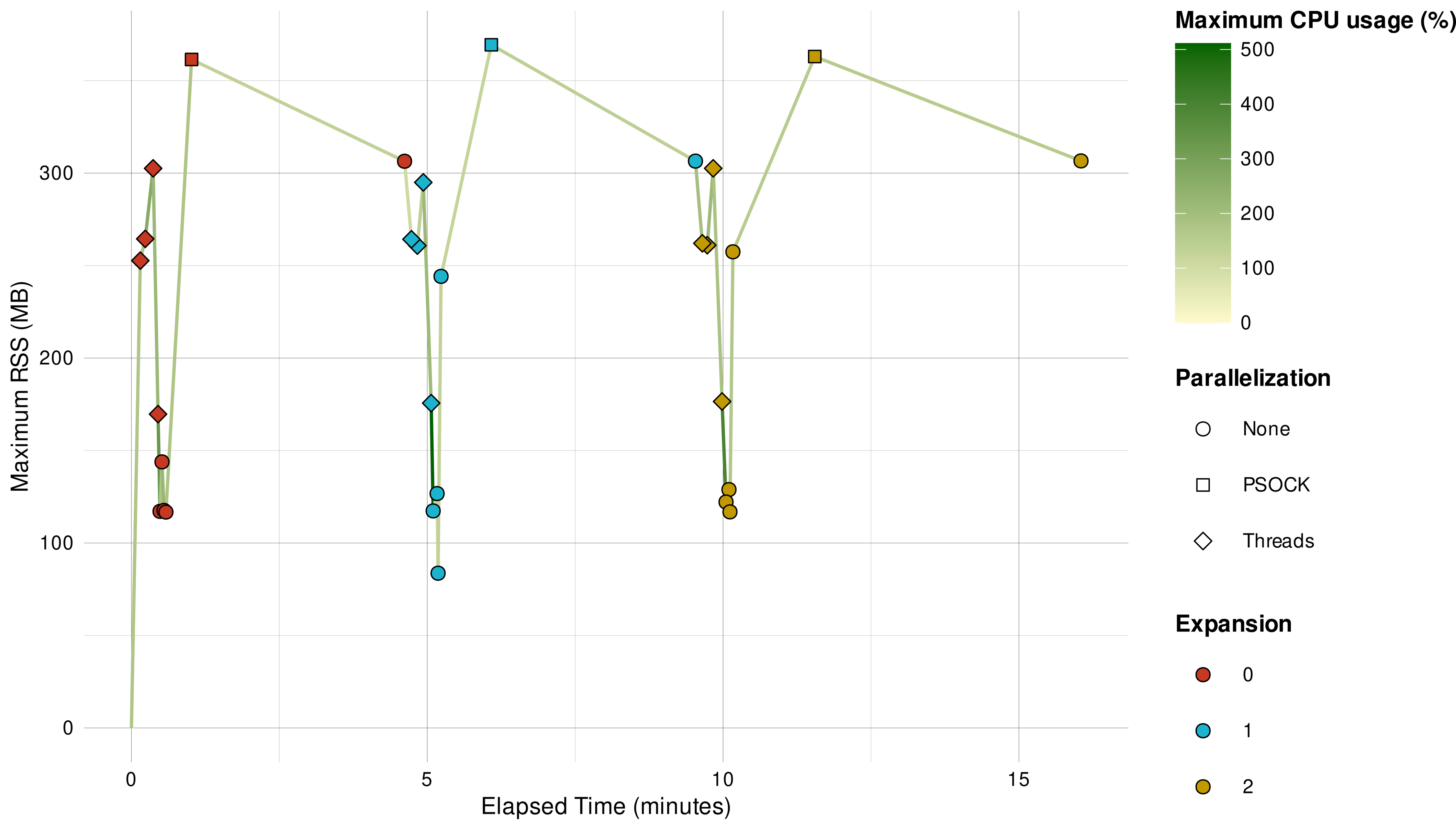

Supplement: S2 Fig — The chart shows the performance monitoring of the example ENQUIRE run described in Results and Fig 2, in which 2 expansions for a total of three iterations were performed. We used a Linux computer with 8 CPUs (2.5 GHz) and 16 GB of RAM. 6 cores were used for parallelization. Each dot represents a submodule launched by ENQUIRE, with the elapsed time at which it terminated as x-coordinate, and the maximum registered RAM usage, in the form of Resident Set Size (RSS, in megabytes), as y-coordinate. Cumulative elapsed time at the end of each reconstruction-expansion cycle is indicated. Lines in-between processes are colored by the maximum CPU usage, which is defined as the used CPU time divided by the time the process has been running, in percentage. This estimate does not typically add up to 100%. Higher CPU usage imply higher workload for each of the utilized cores. Resource usage of parallel socket cluster (PSOCK) protocol can be underestimated, as this protocol generates parallel processes whose process identifiers (PIDs) are independent of ENQUIRE’s PID and not monitored. Nevertheless, ENQUIRE restricts the memory usage of PSOCK-based parallel processes, so that their aggregated memory usage is always less than 25% of the available RAM at a given time, possibly reducing the effective number of cores used. (TIF) [file pcbi.1012745.s003.tif]

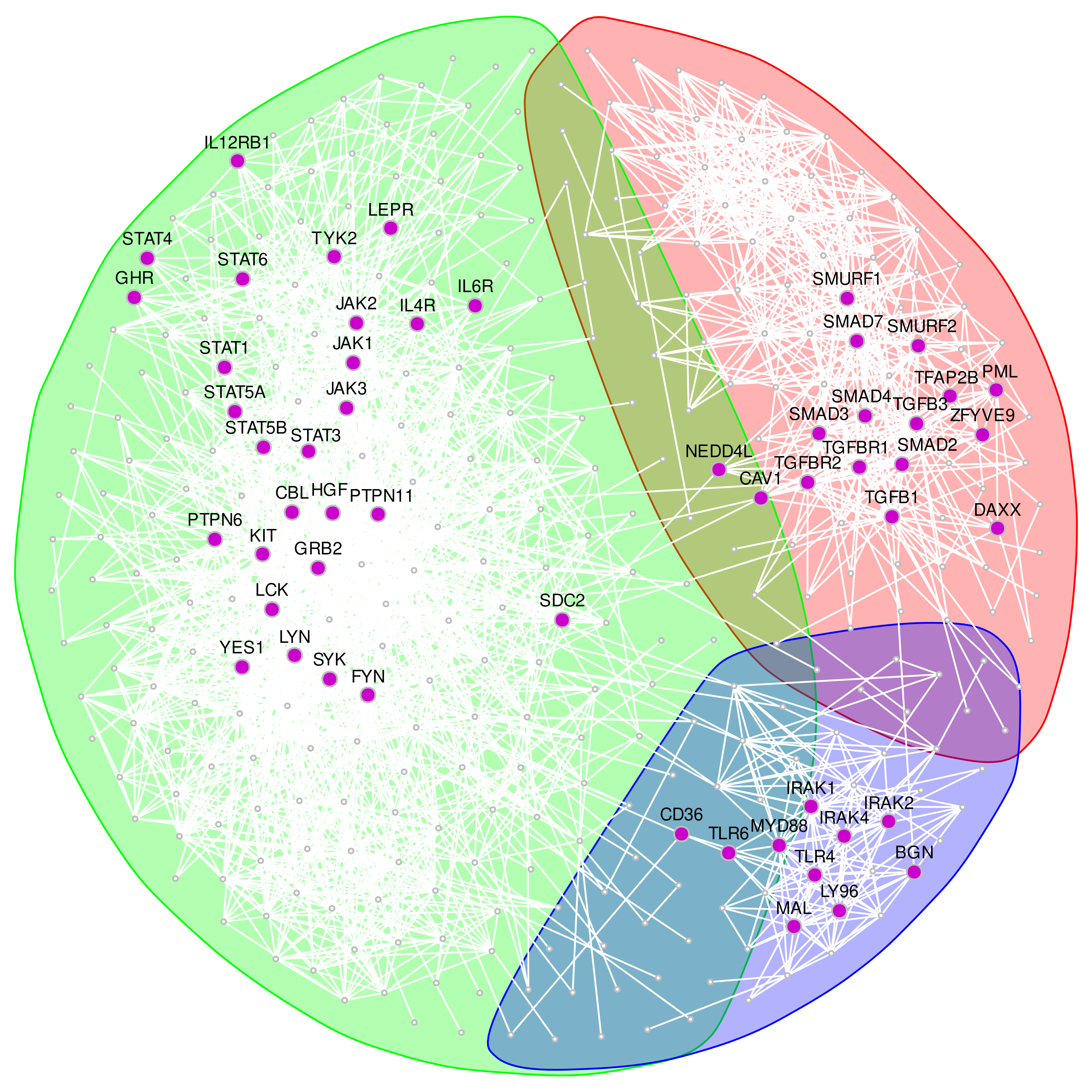

Supplement: S3 Fig — We informed the Q-scored STRING network from ENQUIRE’s context-aware pathway enrichment analysis applied to the non-expanded gene co-occurrence network for the case study Ferroptosis and Immune System. After applying the InfoMap community detection algorithm with normalized Q-scores as probability weights, we selected the three communities with highest average score (colored areas) and pruned the original STRING network using the union of their corresponding protein-coding genes. Nodes with non-zero Q-score are labeled and colored in magenta. (TIF) [file pcbi.1012745.s004.tif]

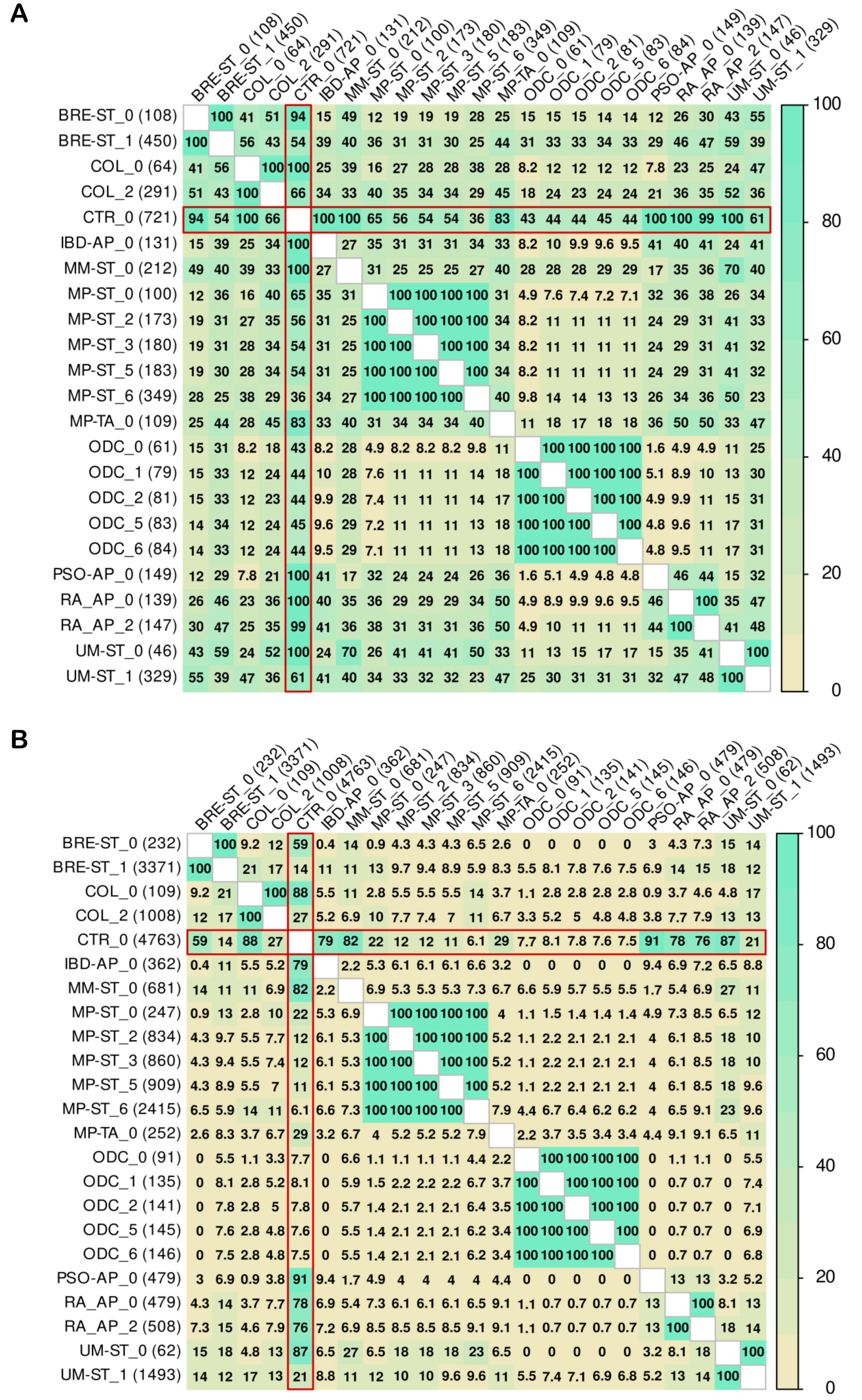

Supplement: S4 Fig — We computed similarity measures between ENQUIRE-inferred, co-occurrence gene networks based on the case studies described in Table 6. The number following a case study abbreviated name indicates the expansion counter. Network expansions that did not yield any new gene were excluded. Panel A depicts similarities between the networks’ node sets, while panel B depicts similarities between edge sets. Numbers and color gradient report Szymkiewicz-Simpson overlap coefficient percentages (OC). An OC of 0% indicates no overlap, while an OC of 100% indicates the smaller node or edge set is a subset of the larger one. By construction, same-case-study original and expanded networks possess OCs of 100% with each other. OC between the positive control (CTR) and other case study networks are highlighted in red. (TIF) [file pcbi.1012745.s005.tif]

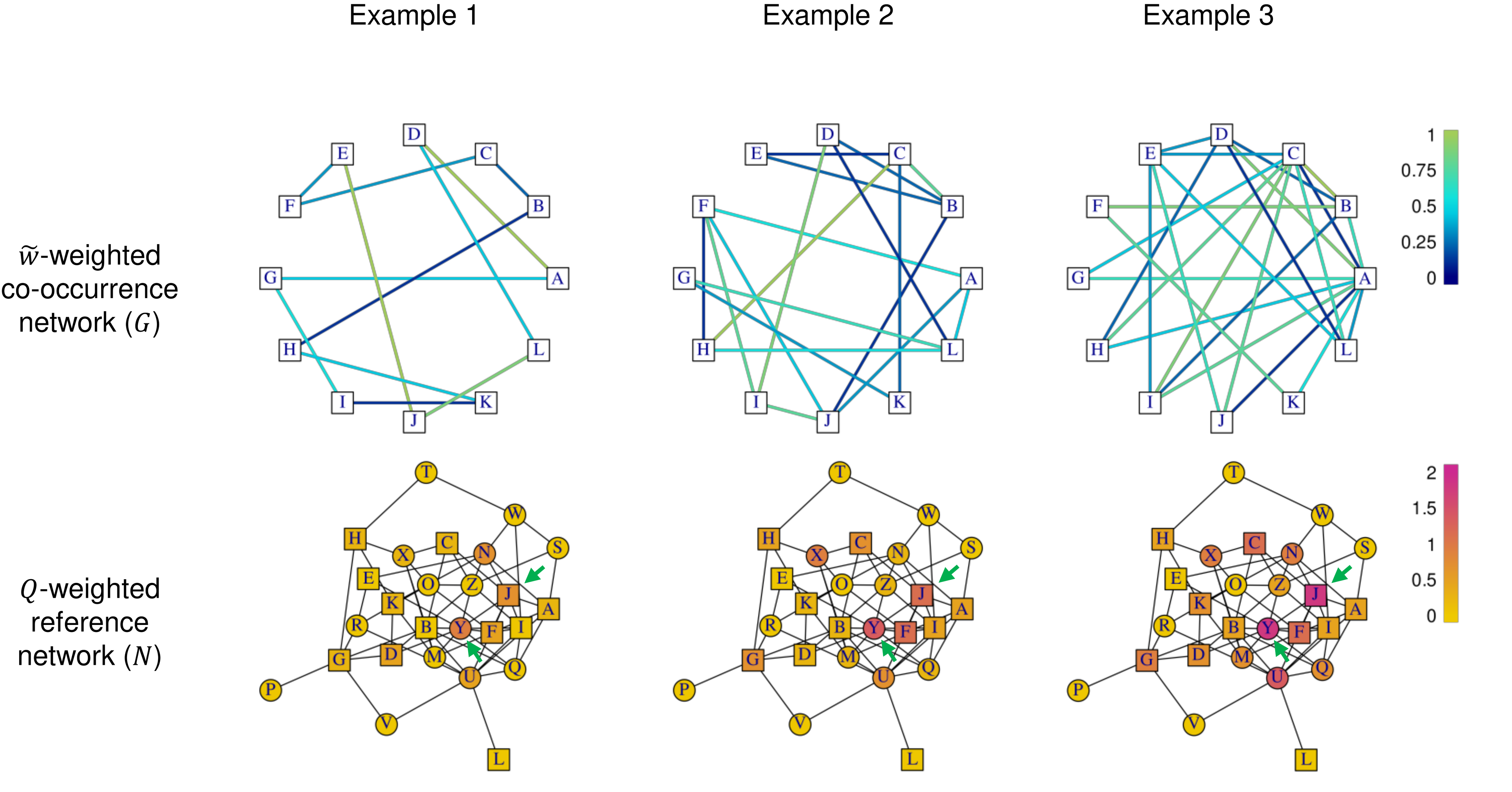

Supplement: S5 Fig — The top row shows three simulated co-occurrence networks G with the same set of text-mined genes (squares), generated with progressively higher edge-forming probability, and sampling edge weights w̃ from a uniform distribution in [0,1]. Genes from an immutable reference network N containing both text-mined and non-text-mined genes (circles) are weighted by the Q score. For each gene g in N, its weight Q is a function of the text-mined genes in the g-neighbourghood and their w̃-weighted distances in the network G. Nodes with relatively more connections to text-mined nodes in the reference network possess higher Q scores, irrespective of being text-mined or having a high node degree. See the non-text-mined node Y and the text-mined node J as an example. (TIF) [file pcbi.1012745.s006.tif]

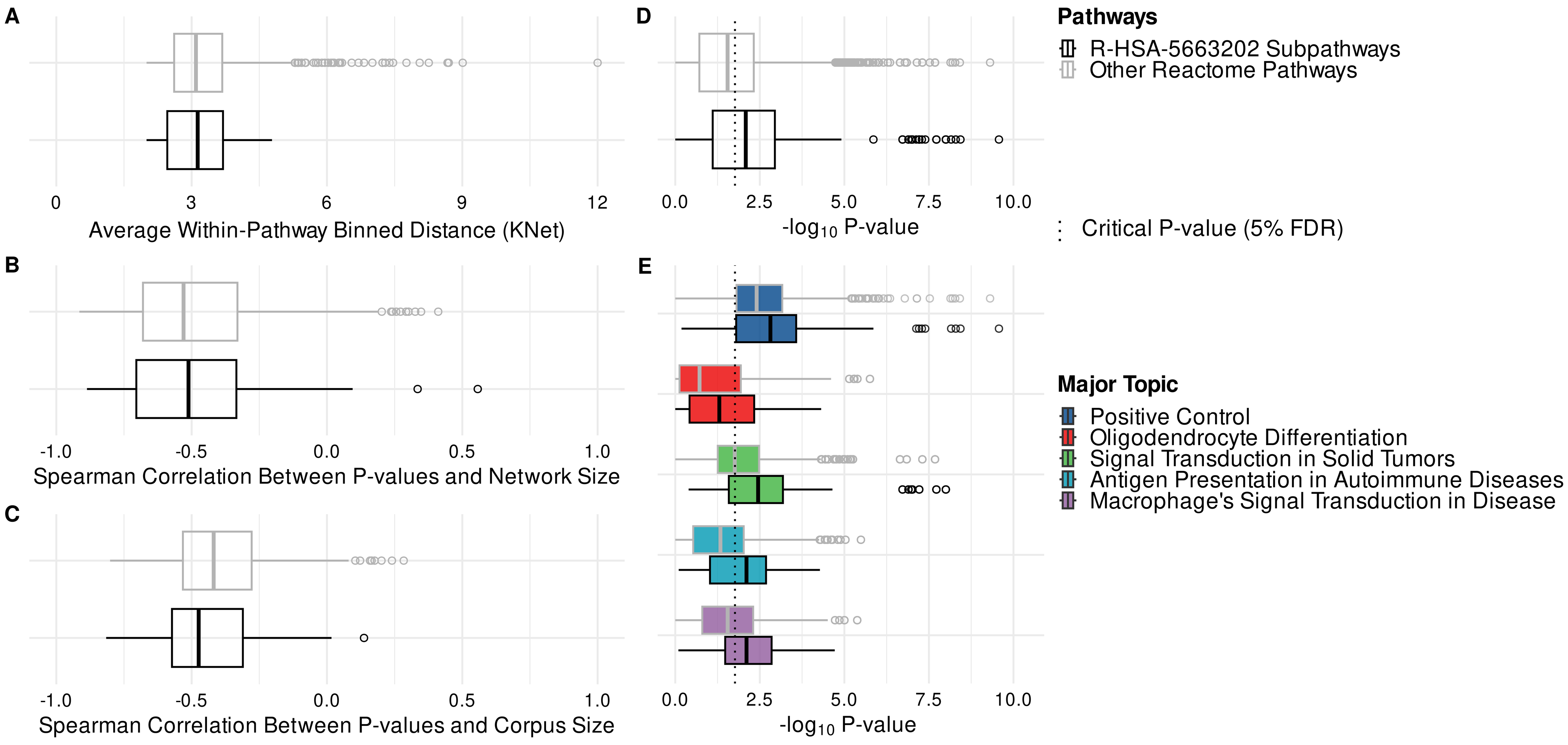

Supplement: S6 Fig — A: differences in network distances between genes belonging to R-HSA-5663202 subpathways and other Reactome pathways, based on STRING’s reference physical network (FDR-adjusted p-value = 0.27, Mann-Whitney U test). The binned network distances are used by KNet to compute a topology-based pathway enrichment. B: differences in Spearman correlations between KNet p-values and network size, in R-HSA-5663202 subpathways and other Reactome pathways (FDR-adjusted p-value = 0.79, Mann-Whitney U test). C: differences in Spearman correlations between KNet p-values and corpus size, in R-HSA-5663202 subpathways and other Reactome pathways (FDR-adjusted p-value = 0.23, Mann-Whitney U test). D: differences in p-value distributions between R-HSA-5663202 subpathways and other pathways, across all case studies (FDR-adjusted p-value = 6.5·10−5, mixed model ANOVA). E: differences in p-value distributions between R-HSA-5663202 subpathways and other pathways, for each major topic (FDR-adjusted p-value (Positive Control) = 0.04– Mann-Whitney U test, FDR-adjusted p-value (Oligodendrocyte Differentiation) = 1.3·10−2, FDR-adjusted p-value (Signal Transduction in Solid Tumors) = 1.4·10−4, FDR-adjusted p-value (Antigen Presentation in Autoimmune Diseases) = 2.3·10−5, FDR-adjusted p-value (Macrophage’s Signal Transduction in Disease) = 3.9·10−4– mixed model ANOVA). See https://zenodo.org/records/12734778 for details on the test statistics. (TIF) [file pcbi.1012745.s007.tif]
