# Supplementary material for: ENQUIRE automatically reconstructs, expands, and drives enrichment analysis of gene and Mesh co-occurrence networks from context-specific biomedical literature
Source: PLoS Comput Biol. 2025 Feb 11;21(2):e1012745. doi: 10.1371/journal.pcbi.1012745 (PMC11844901; doi:10.1371/journal.pcbi.1012745)
Supplement: Attachment — Submitted filename: ENQUIRE.pdf [file pcbi.1012745.s008.pdf]

ENQUIRE (Expanding Networks by Querying Unexpectedly Inter-Related Entities) is an algorithm designed to reconstruct and expand co-occurrence networks of human genes and biomedical ontologies (MeSH) using PubMed articles. The process involves the following steps: the user provides PubMed IDs (PMIDs), the algorithm indexes MeSH terms from these PMIDs and parses the abstracts to identify gene mentions. It annotates and weights the co-occurrences of genes and MeSH terms based on statistical significance. Significant co-occurrences are then used to create an undirected graph. Nodes are weighted, and clusters of high-weight nodes are selected to form network communities. The algorithm identifies optimal sets of community-connecting graphlets, which can be used in additional PubMed queries to find more relevant articles, iteratively expanding the network.

The tool is useful, easy to use, and all the code is freely available. In my opinion, the authors should consider providing an R package instead of a pipeline. Integrating this new type of distribution into the Bioconductor framework would significantly enhance the tool's usability within the omics and biological research community. It would also reduce potential installation issues arising from the pipeline's multiple dependencies on external programs and operating systems.

The paper is well-presented and includes several useful examples; however, it fails to effectively present the data results.
Firstly, the introduction is too generic and strays from the main topic of the paper. It should be more focused on existing tools that work on co-occurrence gene networks, highlighting the differences with ENQUIRE. The knowledge gap that ENQUIRE addresses should be clearly articulated in this section.
The figures lack sufficient detail and exhibit an unacceptable level of resolution quality. For instance, in Figure 2A, it is challenging to discern the differences across the three networks depicted in panel A. It would be helpful if the newly found nodes were labeled with names to facilitate understanding of the data and to acknowledge the annotation effort. Based on the text, it appears that nodes represent genes and MeSH terms; using different node glyphs could enhance readability.

Many figures feature color legends that are larger than the figures themselves. Consider eliminating color code annotations whenever possible, as colors are often difficult to distinguish from each other.

The reader may find it unclear how ENQUIRE contributes to enhancing the biological interpretation of omics data and suggesting relevant processes and components for computational models. While all the results are validated using existing pathways or gene sets, it is not evident how ENQUIRE adds value compared to gene set enrichment alone. The authors should explain and report the results, emphasizing the novel aspects of the networks. For example, they could highlight whether the resulting network reveals new pathways or provides a more comprehensive pathway. It would be interesting to compare ENQUIRE results with Reactome/KEGG gene sets and pathway topology, thereby providing a comparison of gene-gene edges as well.

Co-occurrence networks are known to be susceptible to displaying numerous false or indirect interactions. Providing users with information about the PubMed IDs (PMIDs) that reported specific co-occurrences would be valuable for tracing back all the information if needed. Additionally, having an additional functionality to annotate the final network with existing pathways would be useful for understanding which nodes and edges are supported by existing pathway knowledge and which are novel.

It appears to me that the ENQUIRE approach could be also highly beneficial for updating existing pathways or for generating new pathways, particularly when the studied process is not adequately

represented in existing pathway databases. This application addresses an urgent need within the pathway curation community, and it should be emphasized in the discussion section of the paper.

Minor:
Introduction lines 52-53: You stated “We find this striking, considering the well-documented reproducibility crisis[18–20] his relationship may seem unclear. Could you elaborate more on why you find it striking in relation to the reproducibility crisis?

Introduction line 58-59. “This distinctive element in our methodology allows ENQUIRE to control for literature biases“. The sentence seems too generic, especially considering it's labeled as a "distinctive element." Could you specify which biases you are referring to?

Results: line 76 - You mentioned for the first time "exit conditions" without providing an explanation or referencing a specific part in the text where they are explained. It would be helpful to clarify what these exit conditions entail and provide a more detailed explanation of this aspect here.

Results line 97: You mentioned "together with optional, user-specified parameters" without elaborating on their content or usefulness. It would be beneficial to provide a clearer explanation of what these parameters entail and how they can be utilized by users. Consider explaining this aspect in more detail to enhance understanding.

Results: line 129 The term "attempts A" needs clarification and better explanation. It could refer to the number of times a particular task or process is attempted, but without further context, it's difficult to determine its precise meaning. Consider rephrasing and providing more context to clarify its meaning.

Results line 137: "Non-trivial and descriptive gene sets" could refer to gene sets that are not overly simplistic and accurately describe biological processes or functions. To avoid subjective descriptions, you could rephrase it as "gene sets that capture meaningful and biologically relevant associations."

Discussion line 383: “ENQUIRE interrelates gene mentions and biomedical concepts through co-occurrence networks and tabulated references while accounting for biases in the input literature corpus.” it's not clear how these biases are specifically addressed or whether they can be entirely avoided. It's possible that these biases are inherent in the data used to define and expand the network. More information is needed to clarify this aspect.
