# Supplementary material for: ENQUIRE automatically reconstructs, expands, and drives enrichment analysis of gene and Mesh co-occurrence networks from context-specific biomedical literature
Source: PLoS Comput Biol. 2025 Feb 11;21(2):e1012745. doi: 10.1371/journal.pcbi.1012745 (PMC11844901; doi:10.1371/journal.pcbi.1012745)
Supplement: Attachment — Submitted filename: ENQUIRE_revision_reviewercomments_final.pdf [file pcbi.1012745.s009.pdf]

Dear Prof. Finley and Prof. Ouzounis,

Dear Editorial Office of *PLOS Computational Biology*,

Thank you very much for encouraging us to resubmit our revised manuscript *“ENQUIRE automatically reconstructs, expands, and drives enrichment analysis of gene and MeSH co-occurrence networks from context-specific biomedical literature”*. We are glad that the reviewers found that our approach advances the interpretation of biological networks and that the software is easy to deploy and beneficial, especially within the molecular pathway curation community. However, they also made us aware that more context is necessary to better communicate the importance and utility of the software, while also show its differences to other approaches in more detail, especially in the frame of co-occurrence network reconstruction.

We specifically addressed these concerns by both revising the manuscript and working on ENQUIRE’s software. In particular, the latter effort was mainly directed at making ENQUIRE easy to deploy in scenarios such as the prediction of novel or more comprehensive pathways. For example, we included a statistical test to compare ENQUIRE-reconstructed gene sets to pre-annotated ones and provided the users the possibility to generate a physical interaction network with weights corresponding to ENQUIRE-derived gene-gene co-occurrences, which can be further analyzed with network pruning and community detection algorithms. While revising the manuscript, we also worked to improve the overall clarity of the manuscript, correcting confusing or ambiguous sentences. We feel that the reviewers’ comments and the additional work performed by us has significantly strengthened both the software and the manuscript and we are happy to resubmit our draft.

Please find a detailed point-by-point reply below.

With Best Regards,

Luca Musella and Julio Vera

Reviewer #1:

Review Summary

Q1: "[…] The manuscript is primarily methodological, so I suggest that the Methods section directly follows the Introduction. […]"

Thank you for this suggestion, which fits well to our manuscript's aim to present a novel computational methodology.

PLoS Computational Biology's Author Guidelines recommend for Results to directly follow the Introduction: https://journals.plos.org/ploscompbiol/s/submission-guidelines#loc-parts-of-a-submission.

To reconcile these recommendations, we opted for dedicating more space to ENQUIRE's essential scope and methodology in the Results section's first paragraph (ll. 144-222).

We are, however, open to putting Materials and Methods after Introduction and would like to ask the editors for a final decision on the matter.

Major Issues

Q2: "Dictionary-based extraction of gene names and symbols from free abstract texts can have a significant impact on the quality of downstream tasks' results. Some time ago, PubMed introduced the "Other Term" field, and it would be useful to include this as background knowledge as well".

Thank you for suggesting this mining strategy, which we did not originally take into consideration. During our investigation of the National Library of Medicine's help resources, we learned that the "[tiab]" (Title/Abstract) field also implies querying the "Other Term" field (OT) for the searched term. ENQUIRE postpend "[tiab]" to aliases of a searched gene when generating PubMed queries to expand the network. However, we realized that after fetching the results, the content of the OT was not processed further by ENQUIRE. We have now remedied this in ENQUIRE's code and clarified it in the Methods section (ll. 156-161, 627-629, 800-802).

This measure appears to have improved the recall of relevant genes studied in the textmined articles. For example, the revised version of ENQUIRE detected an additional gene, IL6, in the first co-occurrence network reconstruction for the example case study "Ferroptosis and Immune System" (**Fig. 2** and **Supp. Information**). This is because the gene alias "IL-6" mentioned in PMID 34991659's abstract within "[…] IL-6-TGF-β1 axis […]" was not picked for the reasons explained in **Mat.Met.** (lines ll. 665-670), but was correctly recognized in the OT "TGF-β1/SMAD3/HLF/IL-6/JAK2/STAT3 pathway". To further provide context to the readers in terms of pros and cons of dictionary-based extraction of gene names, we explicitly report precision and recall of the state-of-the-art deep learning model *GNorm2* we used as benchmark at lines ll. 355-356.

Q3: "[…] I missed one or two paragraphs about previous works and state-of-the-art approaches. […] As per the definition, an edge in a co-occurrence network signifies the strength of linkage between two knowledge concepts (i.e., MeSH terms), whereas an edge in a semantic network reveals the nature of the relationship (e.g., "MeSH Term 1 (e.g., substance) inhibits/stimulates Gene A," where the MeSH term could potentially indicate substance). I do not expect to reframe the proposed approach, but the manuscript would benefit from discussing the benefits and limitations of the presented co-occurrence-based approach in relation to more novel, knowledge-based approaches with labels on edges. Please add a paragraph or two to describe state-of-the-art approaches"

We agree with this suggestion. After careful bibliographic review, we discuss usage scenarios of co-occurrence networks and semantic networks at lines ll. 79-112.

Reviewer #2

Major Issues

Q1: "The tool is useful, easy to use, and all the code is freely available. In my opinion, the authors should consider providing an R package instead of a pipeline. Integrating this new type of distribution into the Bioconductor framework would significantly enhance the tool's usability within the omics and biological research community. It would also reduce potential installation issues arising from the pipeline's multiple dependencies on external programs and operating systems"

Thank you very much for your comments on ENQUIRE's usefulness and ease of use, and for suggesting an alternative software distribution to enhance its usability within the biological research community. We are aware that the Bioconductor framework would be compelling for making ENQUIRE's portability and installation easier. However, a scenario where the Bioconductor installation manager would take care of ENQUIRE's dependencies, which consist of several C, Python, and R libraries, could potentially expose the software to errors related to dependency incompatibilities, such as deprecation of necessary package versions. For this reason, we think that a containerization strategy that does not require dependency installation is necessary to ensure compatibility and stability over time. In our first manuscript version, we only offered a Singularity Image File (SIF) as a containerized version of the ENQUIRE software. While we still consider Apptainer/Singularity a very effective and portable solution, it has a clear limitation in it being only Linux-native. Therefore, we decided to also provide a Docker image in order to address the concerns on the usability of ENQUIRE.

Docker is a widely used platform for the deployment of applications and allows for the creation of virtual environments called containers, which act as stand-alone packages that contain all dependencies needed for the applications to run. ENQUIRE users can now simply execute the Docker image, which will initialize a container with all the code and dependencies necessary for ENQUIRE to run, without further concerns for the compatibility of ENQUIRE's source code with their own setup. Detailed instructions specific to the *dockerized* version of ENQUIRE have been included in the GitHub repository, in addition to the previous instructions for the SIF image. The Docker image required to build the ENQUIRE container has been uploaded to Docker Hub.

Docker can be easily installed in Windows, Macintosh and Linux-based systems, and comes with a user-friendly interface known as Docker Desktop. We are confident this solution will make ENQUIRE more portable and accessible to a wider range of users.

Q2: "[…] Firstly, the introduction is too generic and strays from the main topic of the paper. It should be more focused on existing tools that work on co-occurrence gene networks, highlighting the differences with ENQUIRE. The knowledge gap that ENQUIRE addresses should be clearly articulated in this section"

We have followed your suggestion and included a paragraph on differences between ENQUIRE and existing tools for gene co-occurrence networks at lines ll. 79-103 in the Introduction.

Q3: "The figures lack sufficient detail and exhibit an unacceptable level of resolution quality. For instance, in Figure 2A, it is challenging to discern the differences across the three networks depicted in panel A. It would be helpful if the newly found nodes were labeled with names to facilitate understanding of the data and to acknowledge the annotation effort. Based on the text, it appears that nodes represent genes and MeSH terms; using different node glyphs could enhance readability. Many figures feature color legends that are larger than the figures themselves. Consider eliminating color code annotations whenever possible, as colors are often difficult to distinguish from each other"

We regret the insufficient detail and resolution quality of the figures from our initial submission. Firstly, thanks to your suggestion, we reworked **Fig. 2** and separated it into two distinct figures. For **Fig. 2A**, which is now **Fig. 2**, we worked to improve contrast between previously and newly found nodes and edges; we paid attention to better represent connected component structure, edge density, and the role newly found connections have on it; we used different node glyphs to represent genes and MeSH terms, and we added a minimal legend. However, we think that labelling every newly found node would lead to excessive clutter and readability issues. To prevent label cluttering, we only labelled nodes used by ENQUIRE to build PubMed queries, indicated such nodes with a different color on the corresponding expanded networks, and grouped their labels by query to show their impact on the network expansion. We commented on the latter in lines ll. 236-241. We intuited that the most problematic color legends are those involving categorical variables like gene set descriptions (**Fig. 2B**, now **Fig. 3A**) and Reactome pathway category (**Fig. 2C**, now **Fig. 3B**, and **Fig. 5**, now **Fig. 6**). For the former, we opted for simplifying the color legend by only indicating reconstructed gene sets with size bigger or equal to three, reducing the number of colors from eleven to nine and making the remainder ones more distinct between each other. For the latter, we simply removed the color legend and replaced it with direct annotations on the side of the heatmaps. We also generally tried to improve the contrast between different colors in figures with color legends and gradients, see for example "Case Study" color legend and gradients in **Fig. 6**. Lastly, as recommended by the editors, we fed all figures into the Preflight Analysis and Conversion Engine (PACE) digital diagnostic tool, downloaded the resulting TIFF files, and attached them as separate files for the resubmission. They can be downloaded in high-resolution quality in the upper-right corner of the page where the figure appears.

Q4.1: “The reader may find it unclear how ENQUIRE contributes to enhancing the biological interpretation of omics data and suggesting relevant processes and components for computational models. While all the results are validated using existing pathways or gene sets, it is not evident how ENQUIRE adds value compared to gene set enrichment alone. The authors should explain and report the results, emphasizing the novel aspects of the networks. For example, they could highlight whether the resulting network reveals new pathways or provides a more comprehensive pathway […]”

Thank you for suggesting additional ways to scrutinize how ENQUIRE can add value compared to standard procedures in omics data analysis. Alternative to enrichment analysis on pre-annotated, general purpose gene sets and pathways, ENQUIRE can be used to perform *post hoc* analysis on textmined co-occurrence networks that return context-specific gene sets and weighted reference networks associated to the input literature corpus. The scope of the input corpus can be calibrated to closely match an omics experimental setup or a computational model. For example, differentially expressed genes detected in a transcriptomics study could be first queried together with compelling biomedical concepts to generate an input literature corpus for ENQUIRE. We mention these potential applications in the Discussion at lines ll. 564-571. To make ENQUIRE easier to deploy in this context, we have added further functionalities to the respective modules that reconstruct fuzzy gene set clusters and perform context-aware pathway enrichment. In the gene set reconstruction module, we now test if a significant overlap between a reconstructed gene set and a Reactome, *H. sapiens* pathway exists (ORA), using the set of ENQUIRE-derived genes as universe (see lines 800-803 in **Mat.Met.**). We used the algorithm in the example case study *“Ferroptosis and Immune System”* and compared its outcome to standard ORA applied to the entire ENQUIRE-derived set of genes, offered by the STRING database (lines 225-240). For the context-aware pathway enrichment module, we now provide the user with the Q-score-weighted STRING network in the standardized GraphML format as an additional output. Since the Q-score is a function of ENQUIRE-derived gene co-occurrences in the input literature corpus, this network can be pruned or partitioned by third-party algorithms to reveal potentially new or more comprehensive pathways. We provide a proof of concept applying the *InfoMap* community detection algorithm to the Q-score-weighted network obtained from the non-expanded co-occurrence network of *“Ferroptosis and Immune System”* – see lines 249-269, **Supp. Fig. X**, and **Supp. Information**. We further discuss current limitations and future directions in pathway discovery using ENQUIRE in Discussion (lines 556-562).

Q4.2: “[…] It would be interesting to compare ENQUIRE results with Reactome/KEGG gene sets and pathway topology, thereby providing a comparison of gene-gene edges as well.”

We agree that topological comparisons can generate insights unavailable from methods that do not account for gene-gene edges. Please see the below paragraphs for a discussion.

In the submitted version of the manuscript, motivated by comparing co-occurrences derived from literature textmining with non-textmined interactions, we performed a topological comparison on gene-gene edges using STRING-derived, high-confidence

functional interactions restricted to experimental and database evidence. Through STRING, Reactome and KEGG pathways are included in this comparison (see lines ll. 884-907 in Methods for details). We used the DeltaCon distance metric to topologically compare ENQUIRE and STRING networks (**Fig. 4B**, now **Fig. 5B**). We think the about-equal proportion of significantly and non-significantly high similarities between ENQUIRE- and STRING-derived networks indicates that the generated results reflect both known, high-confidence interrelations and novel, previously unannotated ones.

In the dataset using pathway-literature correspondences extracted from Reactome and used to benchmark node weights (**Fig. 3**, now **Fig. 4**), the experimental setup may invite a topological comparison. We think this is not ideal for two main reasons. Firstly, abstracts from Reactome-annotated corpora appear to contain sparse gene mentions that do not reflect the entirety of the genes in the pathway, resulting in low-density, relatively small ENQUIRE-derived co-occurrence networks. This sparsity does not seem to be attributable to ENQUIRE's dictionary-based gene mention extraction as similar results were observed using GNormPlus (**Fig. R1** below; this benchmark was excluded from the manuscript due to the recent release of GNorm2 and replaced by **Tables 1-2**). Secondly, and more importantly in our eyes, we do not intend to claim that text-mined gene co-occurrences should be used as proxies or substitutes for experimentally verified, database-annotated interactions. Instead, we think ENQUIRE's capabilities can help generate context-specific metadata to inform *post hoc* mining strategies that can construct context-specific interactions, gene sets, and pathways from context-unspecific databases and networks. We elaborated on this in lines 98-104 and 536-543 in Introduction and Discussion sections.

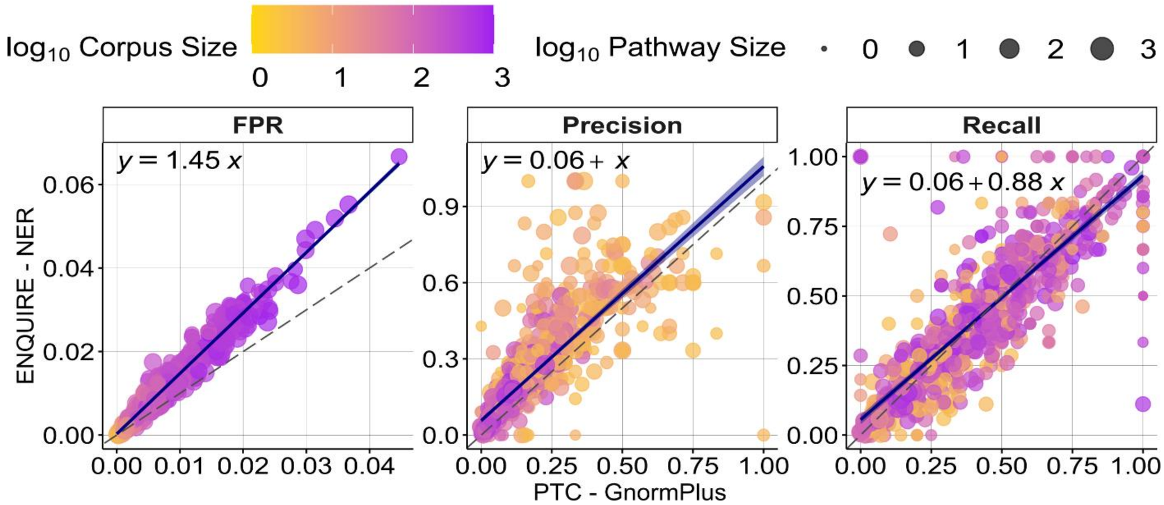

Figure R1. The scatter plots compare ENQUIRE's named entity recognition (NER) of gene mentions to PubTatorCentral's (PTC) implementation of GNormPlus. In each panel, a data point represents one textmining attempt of a set of genes annotated under a Reactome pathway, using its reference literature abstracts as input. The coordinates refer to false positive rate (FPR), precision and recall values independently computed for each tested methodology and pathway. We calculated linear regressions to compare the performances in both panels. We indicated input corpus and pathway size by adjusting color and dot size, respectively. The solid line represents the fitted model obtained from linear regression, with dark grey areas indicating confidence

intervals, while the dashed grey line indicates equality between the two axis values, as a reference.

Q5: "Co-occurrence networks are known to be susceptible to displaying numerous false or indirect interactions. Providing users with information about the PubMed IDs (PMIDs) that reported specific co-occurrences would be valuable for tracing back all the information if needed. Additionally, having an additional functionality to annotate the final network with existing pathways would be useful for understanding which nodes and edges are supported by existing pathway knowledge and which are novel"

We agree that reporting the PMIDs is truly valuable and also necessary. In the original submission, PubMed IDs could be traced back using a dedicated edge table containing any PMID that reports a significant co-occurrence. We have expanded this aspect in the Results section dedicated to the algorithm description – see lines ll. 215-221. Additionally, ENQUIRE now reports information on supporting PMIDs in the corresponding edge and node tables for gene/MeSH, gene-gene, and MeSH-MeSH subgraphs. Similarly, we used the Reactome pathway database to specifically annotate co-occurring genes supported by existing pathway knowledge in the output node and edge tables, as now described in lines ll. 215-218.

Q6: "It appears to me that the ENQUIRE approach could be also highly beneficial for updating existing pathways or for generating new pathways, particularly when the studied process is not adequately represented in existing pathway databases. This application addresses an urgent need within the pathway curation community, and it should be emphasized in the discussion section of the paper"

We thank the reviewer for highlighting one of ENQUIRE's potential applications. Motivated by the previous comments (Q4.1), we used the example case study presented in the manuscript to show how ENQUIRE can inform further analyses that could also lead to updates in existing pathways or novel pathway prediction. However, we acknowledge that further research is needed to develop a systematic framework for pathway discovery, and we enriched the Discussion with future perspectives (ll. 584-590).

Minor Issues

Q7: << Introduction lines 52-53: You stated We find this striking, considering the well-documented reproducibility crisis18–20 this relationship may seem unclear. Could you elaborate more on why you find it striking in relation to the reproducibility crisis?" >>

We are grateful for pointing out a potentially confusing sentence. We rewrote this sentence (lines ll. 100-103) as below:

<< We find this striking, as it's been established that observational studies need to be aggregated to produce stronger scientific evidence, especially in medicine, and that a finding in any such study may turn out to be irreproducible >>.

Q8: << Introduction line 58-59. “This distinctive element in our methodology allows ENQUIRE to control for literature biases”. The sentence seems too generic, especially considering it's labeled as a "distinctive element." Could you specify which biases you are referring to? >>

We agree and rewrote the sentence according to your suggestion at lines ll. 116-118 as below:

<< A distinctive element in our approach is a probabilistic framework aimed at overcoming the limitations of purely bibliometric test statistics, while still accounting for biases generated by uneven representations of biomedical entities in the literature corpus >>

Q9: << Results: line 76 - You mentioned for the first time "exit conditions" without providing an explanation or referencing a specific part in the text where they are explained. It would be helpful to clarify what these exit conditions entail and provide a more detailed explanation of this aspect here >>

We addressed this lack of clarity on the algorithm’s key aspects by adding a more detailed description of the exit conditions immediately next to the modules in which they are evaluated at lines ll 128-162, therefore moving backwards their first mention and in parallel to **Fig. 1**.

Q10: << Results line 97: You mentioned "together with optional, user-specified parameters" without elaborating on their content or usefulness. It would be beneficial to provide a clearer explanation of what these parameters entail and how they can be utilized by users. Consider explaining this aspect in more detail to enhance understanding >>

We removed the mention to such parameters in the caption of **Fig. 1** and improved their description in the paragraph that immediately follows (lines ll. 195-211), together with a mention to the **Mat.Met.** section, **S1 Fig.**, and the GitHub repository (**Deposited Data**) for further details.

Q11: << Results: line 129 The term "attempts A" needs clarification and better explanation. It could refer to the number of times a particular task or process is attempted, but without further context, it's difficult to determine its precise meaning. Consider rephrasing and providing more context to clarify its meaning. >>

We agree and, in line with Q10, added a better explanation for the user-specified parameter A at lines ll. 202-205.

Q12: << Results line 137: "Non-trivial and descriptive gene sets" could refer to gene sets that are not overly simplistic and accurately describe biological processes or functions. To avoid subjective descriptions, you could rephrase it as "gene sets that capture meaningful and biologically relevant associations." >>

We are grateful for the suggested rephrasing; we find it impactful and true to the intended meaning of our original statement and inserted it at lines ll. 253-254.

Q13: << Discussion line 383: "ENQUIRE interrelates gene mentions and biomedical concepts through co-occurrence networks and tabulated references while accounting for biases in the input literature corpus." it's not clear how these biases are specifically addressed or whether they can be entirely avoided. It's possible that these biases are inherent in the data used to define and expand the network. More information is needed to clarify this aspect >>

Indeed, we did not fully clarify the biases we refer to in this sentence, which relate to similar concerns as Q8. We therefore refer again to the revised Introduction (lines ll. 116-118) and also clarified that we expect inherent biases in the data that we try to correct for with the proposed statistical framework (lines ll. 546-549):

<< ENQUIRE interrelates gene and MeSH mentions through co-occurrence networks and tabulated references via a probabilistic framework that accounts for the bias of uneven representation of entities in the input literature corpus, without being dependent on the corpus size >>
